# A fatal case of fulminant neuroleptic malignant syndrome: A case report

**DOI:** 10.1002/pcn5.75

**Published:** 2023-01-23

**Authors:** Hitomi Komoriya, Konoka Nomura, Toshinori Shirata, Tadahiro Kobayashi, Masaki Nakane, Keisuke Noto, Ryota Kobayashi, Akihito Suzuki

**Affiliations:** ^1^ Department of Psychiatry Yamagata University School of Medicine Yamagata Japan; ^2^ Department of Emergency and Critical Care Medicine Yamagata University School of Medicine Yamagata Japan

**Keywords:** fatal and fulminant case, neuroleptic malignant syndrome, schizoaffective disorder, severe autonomic disturbances, severe hyperthermia

## Abstract

**Background:**

Neuroleptic malignant syndrome (NMS), a rare but potentially life‐threatening adverse reaction to treatment with antipsychotic drugs, is characterized by hyperthermia, muscle rigidity, impaired consciousness, and autonomic disturbances. Some reports have described rapidly progressing cases of NMS resulting in death within several days. This report describes a clinical course of fatal and fulminant NMS in a patient with schizoaffective disorder.

**Case Presentation:**

A 67‐year‐old man had long been in a stable condition under antipsychotic pharmacotherapy. At 3 days before admission to our hospital, he complained of diarrhea, fatigue, and reduced appetite. On admission to our hospital, he showed fever, mild muscle rigidity at the four extremities, elevated heart rate, hypertension, excessive diaphoresis, and decreased percutaneous oxygen saturation (SpO_2_). He was diagnosed as having NMS. Within 3 days after the onset of NMS, he displayed severe hyperthermia up to 41.4°C and severe autonomic disturbances, including elevated heart rate and hypertension. Despite treatments with dantrolene and bromocriptine, he went into shock and died on the fourth day after admission.

**Conclusion:**

The present case suggests that severe hyperthermia and severe autonomic disturbances at the early stage of the onset might be signs of fatal and fulminant NMS. It may be recommended that clinicians consider electro‐convulsive therapy when treating fulminant NMS with these symptoms.

## BACKGROUND

Neuroleptic malignant syndrome (NMS), a rare adverse reaction of antipsychotic treatment, is characterized by hyperthermia, muscle rigidity, impaired consciousness, and autonomic nervous disturbances.^1^, ^2^, ^3^ This potentially life‐threatening syndrome has mortality rates ranging from 5% to 22%.^2^ NMS has several risk factors reported as contributing to mortality: severity of hyperthermia, respiratory alterations, and older age.^3^ Nevertheless, few reports have described the rapidly progressing course of this syndrome, which can lead to death within several days.^4^, ^5^, ^6^, ^7^, ^8^ This report describes a fatal case of fulminant NMS in a patient with schizoaffective disorder.

## CASE PRESENTATION

This case is that of a 67‐year‐old man with schizoaffective disorder. He had developed auditory hallucinations, persecutory delusions, and depressive mood in his 50s, and had been diagnosed with schizoaffective disorder at a psychiatric hospital. His condition had long been stable at least for 10 years under treatment with 12.5 mg/day olanzapine, 2 mg/day biperiden, 20 mg/day promethazine, and 3 mg/day diazepam. Then he had lived alone independently. He had also been administered 40 mg/day valsartan and 2.5 mg/day amlodipine as treatments of hypertension, and 48 μg/day lubiprostone and 150 mg/day aclatonium napadisilate for constipation. He had never drunk alcohol. Written informed consent for reporting his clinical course was obtained from him when he was in a state of clear consciousness on admission to our hospital. This report was approved by the Ethical Review Committee of Yamagata University Faculty of Medicine.

At 3 days before admission to our hospital, he complained of diarrhea, fatigue, and reduced appetite. He visited the psychiatric hospital, where he displayed a pale complexion, diaphoresis, and decreased percutaneous oxygen saturation (SpO_2_) at 91% and therefore he was transported by ambulance to the emergency department of our hospital. On admission, he was lucid, with 37.7°C body temperature, 120–130 beats/min heart rate (HR), 30/min respiratory rate (RR), and SpO_2_ was the first half of 90% with nasal oxygen at 4 l/min. He showed mild muscle rigidity in the four extremities and excessive diaphoresis. Laboratory blood tests showed increased levels of creatine kinase (CK) (9811 U/l), aspartate aminotransferase (AST) (256 U/l), lactate dehydrogenase (514 U/l), and white blood cells (12,260/μl). In an arterial blood gas analysis, oxygen partial pressure, carbon dioxide partial pressure, potential of hydrogen, and the ratio between arterial oxygen partial pressure and fraction of inspired oxygen were 102.0, 35.4 mmHg, 7.432, and 280, respectively, suggesting good oxygenation. Chest X‐ray, contrast‐enhanced whole‐body computed tomography (CT) scan, and cardiac and abdominal ultrasound showed no abnormality. The blood concentration of procalcitonin was 0.04 ng/ml. Pathogens of common bacteria, mycobacterium, and fungi were examined in blood, urine, sputum, and nasal mucosa sputum, with no evidence indicating infection of the central nervous system or other organs. According to the criteria of Caroff and colleagues,^1^ he was diagnosed as having NMS, possibly triggered by dehydration because of diarrhea under the antipsychotic treatment. Treatment with 40 mg/day dantrolene was started; it was later increased to a dosage of 200 mg/day. Massive infusion (3000 ml/day), cooling, and supportive treatments were applied.

On the second day after the admission, his muscle rigidity had disappeared, but he displayed severe hyperthermia at 41.4°C, increased HR at 131 beats/min, increased RR at 35/min, increased blood pressure at 161/94 mmHg, and impaired consciousness (Glasgow Coma Scale = 11). Blood tests showed increased concentrations of white blood cells (21,400/μl) and blood sugar (175 mg/dl). Tracheal intubation and mechanical ventilation were performed because of respiratory failure. On the third day, he developed myoglobinuria with oliguria (20 ml/h). His severe hyperthermia was mitigated by cooling using the administration of cold water via a nasogastric tube in addition to systemic cooling with an air‐blower. Blood tests showed increased concentrations of CK (19,718 U/l), AST (1219 U/l), and alanine aminotransferase (950 U/l). Bromocriptine 5 mg/day was started. On the fourth day, a whole‐body CT scan, including the brain, was performed again, revealing no abnormality. Findings implying pulmonary infarction, pneumonia, and venous thromboembolism were not found on the chest X‐rays and contrast‐enhanced whole‐body CT scans. Cerebrospinal fluid examination and magnetic resonance imaging were unable to be performed throughout his course because of the rapid deterioration of his general condition. He went into shock and died thereafter. No postmortem examination was performed because his family did not wish to have an autopsy. Based on the findings on examinations and his clinical course, a doctor in the Department of Emergency and Critical Care Medicine determined the cause of death as multiple organ failure, that is, respiratory, liver, and kidney failures, caused by NMS.

## DISCUSSION

The present case had been in stable condition for over 10 years and he had never displayed catatonic symptoms. In addition, his hyperthermia, muscle rigidity, impaired consciousness, and autonomic nervous disturbances emerged under the treatment with olanzapine. Based on these findings in addition to the criteria of Caroff and colleagues,^1^ the diagnosis of NMS was applied to this case. However, the possibility that the diagnosis in the present case was malignant catatonia cannot be excluded entirely because previous reports suggested that clinical characteristics, course, and response to treatment in NMS are indistinguishable from those in malignant catatonia, and that NMS and malignant catatonia reflect the same pathophysiology.^9^

The four core symptoms of hyperthermia, muscle rigidity, impaired consciousness, and autonomic nervous disturbances characterize NMS.^1^, ^2^, ^3^ Velamoor and colleagues^10^ examined the order of occurrence of these symptoms using the case reports of 340 patients with NMS. The findings suggest that either muscle rigidity or impaired consciousness is the initial symptom of NMS. Subsequently, hyperthermia and autonomic disturbances emerge. Furthermore, a systematic review by Guinart and colleagues^3^ suggested that the risk factors of mortality associated with NMS are severity of hyperthermia, autonomic disturbances including respiratory alterations, and older age. In a systematic review, Belvederi Murri and colleagues^11^ compared the order of occurrence of NMS symptoms and the outcome among several second‐generation antipsychotics, and they suggested that treatment with olanzapine, quetiapine, or clozapine was related to the occurrence of autonomic symptoms at the early stage of NMS onset and a fatal outcome, compared with risperidone and aripiprazole.

The present case showed severe hyperthermia as high as 41.4°C and autonomic disturbances including elevated heart rate, hypertension, and decreased SpO_2_ within 3 days after the onset of NMS. Table 1 presents a summary of earlier reports describing fatal cases of fulminant NMS,^4^, ^5^, ^6^, ^7^, ^8^ which is adapted from the criteria for the severity of NMS symptoms.^12^, ^13^ Although some reports have lacked information about the severity of NMS symptoms, the findings of these earlier reported cases show agreement with the findings obtained for the present case in terms of the occurrence of severe hyperthermia and severe autonomic disturbances as well as rigidity and impaired consciousness within a few days after the NMS onset. The emergence of severe hyperthermia and severe autonomic disturbances at the early stage of the onset might therefore be signs of fatal and fulminant NMS. On the other hand, dopamine blockade in the hypothalamus is putatively related to hyperthermia and autonomic disturbances, whereas dopamine blockade in the nigrostriatal pathway and in the brain stem reticular, respectively, cause muscle rigidity and impaired consciousness.^3^, ^14^ Thus, the antipsychotic‐induced dopamine blockade in the hypothalamus might be implicated in the rapidly progressing course of NMS resulting in death.

**Table 1:** Fatal cases of fulminant neuroleptic malignant syndrome.

Reference	Hyperthermia	Autonomic nervous disturbances	Muscle rigidity	Impaired consciousness	CPK	WBC	Treatment
Present case	Severe (41.4°C at maximum on the second day)	Severe (HR 131 bpm, RR 35, BP 167/90 at maximum on the first to second days)	Mild (at maximum on the first day)	Substupor (from the second day onwards)	19,718 U/l at maximum on the third day	22,045/μl at maximum on the third day	Bromocryptine, dantrolene
Zou et al.^8^	Severe (42°C)	Severe (HR 158 bpm, RR 52, BP 120/60)	+ (n.d.d.)	Unconscious	Not reported	26,500/μl	Not reported
Varoglu et al.^7^	Severe (40°C)	− (BP 100/60)	+ (n.d.d.)	Disturbed conciousness	442 IU/l	6100/μl	Bromocryptine, diazepam
Benaissa et al.^6^	Severe (41.2°C)	Severe (HR 148 bpm, RR 34, BP 120/)	+ (n.d.d.)	Unconscious	813,000 IU/l	11,800/μl	Bromocryptine, dantrolene, pancuronium
Teo et al.^5^	+ (n.d.d.)	Mild (HR 98 bpm, RR 20, BP 120/80)	Severe (lead‐pipe rigidity)	Stupor	33,780 U/l	4700/μl	Bromocryptine, dantrolene, phenobarbitone, phenytoin
Lenler‐Petersen et al.^4^	Severe (41°C)	Severe (HR 170 bpm, excessive hyperventilation)	+ (n.d.d.)	Unconscious	3380 U/l	14,600/μl	Diazepam, pancuronium, physostigmine

*Note*: Severity of NMS symptoms is determined according to the criteria of Strawn et al. and Woodbury and Woodbury.^12^, ^13^

Abbreviations: BP, blood pressure; CPK, creatine phospho‐kinase; HR, heart rate; n.d.d., no detailed description; RR, respiratory rate; WBC, white blood cell count.

The present case was treated using dantrolene and bromocriptine, with no apparent improvement, eventually leading to death. Treatment using benzodiazepines, which was reported to relieve the symptoms of NMS,^9^ was not performed in this case because of the risk that his decreased SpO_2_ levels on admission were further exacerbated due to respiratory depression by the drugs. In earlier reported fatal cases of fulminant NMS,^4^, ^5^, ^6^, ^7^, ^8^ dantrolene, bromocriptine, antiepileptics, and muscle relaxants had been used, but all were ineffective (Table 1). Kuhlwilm and colleagues^2^ performed a systematic review particularly addressing the treatments and outcomes of NMS. They suggested that treatments including dantrolene, bromocriptine, and electroconvulsive therapy (ECT) are effective for severe NMS. Furthermore, they suggested that the mortality rate in the ECT is lowest among the treatments. Consequently, it might be recommended that clinicians consider ECT in their treatment of NMS with severe hyperthermia and severe autonomic disturbances at the early stage of onset. Meanwhile, no studies comparing the efficacies for NMS between benzodiazepines and other treatments have been performed. Thus, more such case reports are warranted to elucidate the appropriate treatment of severe NMS.

## CONCLUSION

The present report suggests that severe hyperthermia and severe autonomic disturbances at the early stage of onset might be signs of fatal and fulminant NMS. It may be recommended that clinicians consider ECT when treating fulminant NMS with these symptoms.

## AUTHOR CONTRIBUTIONS

Hitomi Komoriya, Konoka Nomura, Keisuke Noto, Tadahiro Kobayashi, and Masaki Nakane treated the patient and drafted the manuscript. Toshinori Shirata and Ryota Kobayashi critically reviewed the draft and revised it. All authors approved the final version of the manuscript.

## CONFLICT OF INTEREST STATEMENT

The authors declare no conflict of interest.

## ETHICS APPROVAL STATEMENT

Written informed consent for presentation of his clinical course was given by the patient. This report has been approved by the Ethics Review Committee of Yamagata University Faculty of Medicine.

## PATIENT CONSENT STATEMENT

Written informed consent for presentation of his clinical course was given by the patient. This report has been approved by the Ethics Review Committee of Yamagata University Faculty of Medicine.

## CLINICAL TRIAL REGISTRATION

N/A.

## Data Availability

All relevant data are included in the paper.
